# A Multidrug Therapy for Hydrocarbon Aspiration With Acute Respiratory Distress Syndrome After Exposure to Oral Benzine Intake: A Case Report

**DOI:** 10.7759/cureus.19693

**Published:** 2021-11-18

**Authors:** Mari Uno, Takashi Hongo, Sho Kobayashi, Tomokazu Tamura

**Affiliations:** 1 Internal Medicine, Okayama Saiseikai General Hospital, Okayama, JPN; 2 Emergency Medicine, Okayama Saiseikai General Hospital, Okayama, JPN

**Keywords:** ards, hydrocarbon aspiration, sivelestat sodium hydrate, steroid, benzine

## Abstract

Oral benzine intake with suicidal tendencies is an uncommon life-threatening respiratory emergency without a treatment regimen.

A 50-year-old man attempted suicide with 100 ml of oral benzine intake and developed severe acute respiratory distress syndrome (ARDS) with hydrocarbon aspiration. He received mechanical ventilation with placement in the prone position and low tidal volume, neuromuscular blocking agents, bronchoalveolar lavage, steroid pulse therapy, antibiotics, and sivelestat sodium hydrate. He was transferred to the psychiatric hospital five days after admission without any adverse events.

ARDS associated with oral benzine intake could be treated with general treatments for ARDS.

## Introduction

Benzine, a mixture of liquid aliphatic and hydrocarbons, is cheap and readily available in supermarkets or the Internet for use for eradication [1]. Benzine causes significant acute respiratory distress syndrome (ARDS) and is associated with high morbidity and mortality due to volatile agents and hemodynamic instability points [2-4]. Oral benzine intake cases are uncommon without definitive therapies [5]. General treatments for ARDS including steroid pulse therapy and/or sivelestat sodium hydrate have not been established as effective treatments for oral benzine intake.

Here, we report a case of hydrocarbon aspiration with severe ARDS caused by oral benzine intake in a 50-year-old Japanese man treated with supportive care including steroid pulse therapy and/or sivelestat sodium therapy without any complications.

## Case presentation

A 50-year-old Japanese man, previously in good health, presented to our emergency department with progressive shortness of breath, vomiting, and hematuria. His medical history was unremarkable, with no history of mental disorders, smoking, allergy, anaphylaxis, or bronchial asthma. He attempted suicide by ingestion of 900 mg of diphenhydramine and 100 ml of benzine 10 h before arrival. His height was 177 cm, weight 68.0 kg, and his vital signs were as follows: Glasgow Coma Scale score of 12 (E3V3M6); blood pressure, 111/64 mmHg; heart rate, 137 bpm; body temperature, 37.8°C; respiratory rate, 44 breaths/min; and arterial oxygen saturation, 80 with oxygen delivery via a face mask (10 l/min). Physical examination revealed rigorous inspiratory retraction and bilateral polyphonic wheezing. Arterial blood gas analysis showed hypoxemia and laboratory results showed leukocytosis and high plasma D-dimer levels, while other results were unremarkable (Table 1).

**Table 1:** Laboratory data on admission APTT, activated partial thromboplastin time; AST, aspartate aminotransferase; ALT, alanine aminotransferase; ALP, alkaline phosphatase; GGTP, gamma glutamyl transpeptidase; CK, creatinine kinase; LDH, lactate dehydrogenase; BUN, blood urea nitrogen; CRP, C-reactive protein; BAL, bronchoalveolar lavage; BE, base excess; Lac, lactate

Hematologic tests					
White blood cells	29,600 cells/μl			Platelet count	35.7 × 10^4 ^/μl
Neutrophils	91.5%			Prothrombin time	9.6 s
Lymphocytes	4.0%			APTT	22.2 s
Monocytes	4.5%			D-dimer	43.5 μg/ml
Eosinophils	0%				
Basophils	0%				
Red blood cells	650 × 10^4 ^/μl				
Hemoglobin	19.5 g/dl				
Blood biochemistry					
Total protein	6.8 g/dl			LDH	237 U/l
Albumin	4.1 g/dl			BUN	20 mg/dl
Total bilirubin	1.3 mg/dl			Creatinine	0.99 mg/dl
AST	20 U/l			Sodium	141 mEq/l
ALT	20 U/l			Potassium	4.0 mEq/l
ALP	64 U/l			CRP	0.20 mg/dl
GGTP	22 U/l			Glucose	188 mg/dl
CK	68 U/l				
Arterial gas analysis			BAL on 2nd day after admission		
pH	7.428			Cell	39/μl
pO_2 _	45.3 mmHg (10 l/min of oxygen)			Neutrophils	64%
pCO_2_	35.8 mmHg			Macrophage	0%
HCO_3_	19.0 mmol/l			Lymphocytes	17%
BE	-6.2 mmol/l			Alveolar macrophages	13%
Lac	5.2 mmol/l			Bacterial culture	Negative
				Viral	Negative

**Figure 1:**
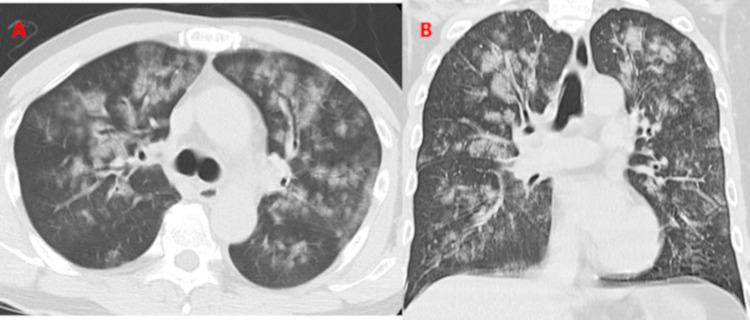
Chest computed tomography on admission Computed tomography revealed diffuse ground-glass opacity in the bilateral lung large (A) axial view (B) coronal view.

Chest radiography upon admission revealed bilateral patchy airspace opacities in the mid and lower lung zones. Chest computed tomography (CT) indicated diffuse ground-glass opacity in both lungs (Figure 1).

**Figure 2:**
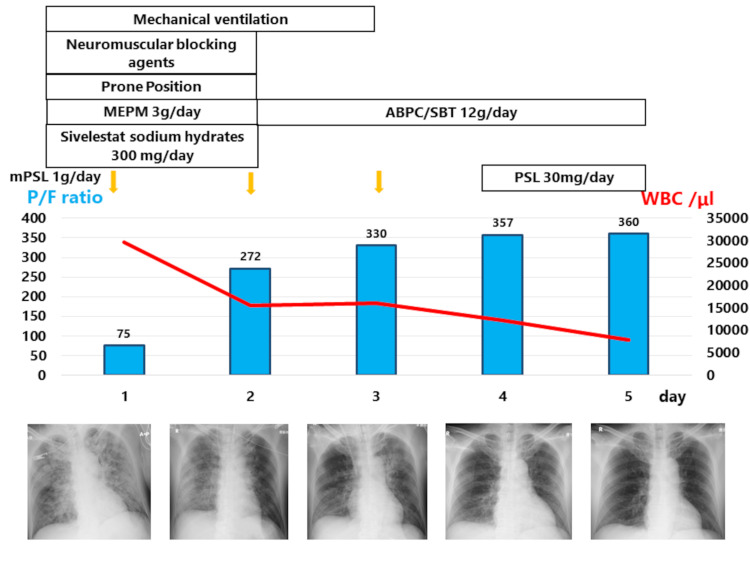
The clinical course of hydrocarbon aspiration with acute respiratory distress syndrome after exposure to oral benzine intake in a 50-year-old Japanese man Mechanical ventilation was needed for 3 days, and neuromuscular blocking agents, antibiotics, steroid therapy, and sivelestat sodium hydrate were administered MEPM, meropenem; mPSL, methylprednisolone; PSL, prednisolone; P/F, PaO_2_/FiO_2_; ABPC/SBT, ampicillin/sulbactam; WBC, white blood cell

He was admitted to the intensive care unit and provided mechanical ventilation in the prone position, neuromuscular blocking agents (rocuronium bromide 7 μg/kg/min), and sedation (propofol 1.0-2.0  mg/kg). The ventilator settings were 6 cc/kg with an increased positive end expiratory pressure of 10 to address ARDS caused by hydrocarbon aspiration. We started intravenous treatment with antibiotics (meropenem 3 g/day), steroid pulse therapy (methylprednisolone 1 g/day), and sivelestat sodium hydrate (300 mg/day). The clinical course is shown in Figure 2. Bronchoalveolar lavage (BAL) was performed on the second day after admission to rule out bacterial infection or fat embolism. BAL revealed lymphocytic alveolitis (39 cells with 64% neutrophils, 0% macrophages, 17% lymphocytes, and 13% alveolar macrophages). Bacterial cultures and viral studies were negative. Upper gastrointestinal tract endoscopy showed no esophageal, gastric, and duodenal bleeding or erosion.

After three days of treatment, the patient’s general condition improved and he was weaned from mechanical ventilation. He was transferred to the psychiatric hospital on the fifth day after admission without any supportive treatments, including the use of oxygen therapy.

## Discussion

Benzine is an organic solvent produced from petroleum, consisting of n-pentane and n-hexane, and quickly evaporates at ambient temperature [1,3]. These characteristics can lead to lung injury, myocardial injury, seizures, kidney injury, and liver injury [1-3]. Hydrocarbon aspiration involving benzine can result in asphyxia, chemical pneumonitis, lipoid pneumonia, pulmonary edema, and hemorrhage. These conditions are common life-threatening disorders with rapid progression. It is caused by the primary effect of chemicals or secondarily by respiratory depression and suffocation due to volatile fluid [6]. Oral benzine intake is uncommonly described, and we are aware of no published reports of hydrocarbon aspiration caused by benzine oral intake.

Current hydrocarbon aspiration therapy is supportive therapy with mechanical ventilation. In addition to supportive care, treatments, including intravenous antibiotics and steroids, are commonly used for hydrocarbon aspiration [5,6]. Although antibiotics are ineffective in treating hydrocarbon pneumonitis, Chen et al. proposed that all cases with hydrocarbon aspiration should be prescribed antibiotics [5]. In this case, antibiotics were used due to significant leukocytosis, which is also a typical laboratory finding in cases of severe bacterial infection.

Steroids are controversial treatments for hydrocarbon aspiration [5]. Animal experiments have not provided convincing evidence that steroids are effective for hydrocarbon aspiration. The evidence for steroid effectiveness is based on reports published in the 1970s [7]. However, several case studies have shown that patients with hydrocarbon pneumonitis improve with steroid therapy [5,8]. We administered steroids plus treatment without any adverse events, similar to the results reported in previous studies [5,8]. Other case reports have noted dramatic improvements after BAL; administration of immunoglobulins, surfactants, and nitric oxide; and hemofiltration in patients with hydrocarbon injection with pneumonitis [1,6].

 We also used sivelestat sodium hydrate to significantly inhibit tumor necrosis factor-α and interleukin-6 production, inducible expression of nitric oxide synthase protein and box 1 of the high mobility group box 1 (HMGB1), and reduce serum nitrite levels [9,10]. Yoshikawa et al. reported that sivelestat sodium hydrate reduced radiation-induced lung injury in mice by inhibiting neutrophil elastase activity and excessive inflammatory reactions [11]. The hydrocarbon aspiration mechanism results from the consumption of the antioxidant defense system with consequential loss of cell and tissue integrity [6]. The shorter hospital stay without adverse events suggests that sivelestat sodium hydrate helps improve the respiratory condition after benzine injection [5]. One of the limitations could be non-generalizability because steroid pulses alone led to remission and additional studies are needed to provide established evidence of the effectiveness of these therapies for benzine poisoning.

## Conclusions

ARDS treatment including the steroids and sivelestat sodium hydrates might affect the favorable outcomes of hydrocarbon aspiration after exposure to oral benzine intake.
